# CircRNAs as potent biomarkers in ovarian cancer: a systematic scoping review

**DOI:** 10.1186/s11658-021-00284-7

**Published:** 2021-09-23

**Authors:** Zahra Foruzandeh, Fatemeh Zeinali-Sehrig, Kazem Nejati, Dara Rahmanpour, Fariba Pashazadeh, Farhad Seif, Mohammad Reza Alivand

**Affiliations:** 1 Department of Medical Genetics, Faculty of Medicine, Tabriz University of Medical Sciences, Tabriz, Iran; 2 Pharmaceutical Sciences Research Center, Ardabil University of Medical Science, Ardabil, Iran; 3 Research Center for Evidence-Based Medicine, Tabriz University of Medical Science, Tabriz, Iran; 4 Department of Immunology and Allergy, Academic Center for Education, Culture, and Research, Tehran, Iran

**Keywords:** Circular RNA (CircRNA), Ovarian cancer (OC)

## Abstract

More powerful prognostic and diagnostic tools are urgently needed for identifying and treating ovarian cancer (OC), which is the most fatal malignancy in women in developed countries. Circular RNAs (circRNAs) are conservative and stable looped molecules that can regulate gene expression by competing with other endogenous microRNA sponges. This discovery provided new insight into novel methods for regulating genes that are involved in many disorders and cancers. This review focuses on the dysregulated expression of circRNAs as well as their diagnostic and prognostic values in OC. We found that studies have identified twenty-one downregulated circRNAs and fifty-seven upregulated ones. The results of these studies confirm that circRNAs might be potent biomarkers with diagnostic, prognostic and therapeutic target value for OC. We also consider the connection between circRNAs and OC cell proliferation, apoptosis, metastasis, and chemotherapy resistance and sensitivity.

**Supplementary Information:**

The online version contains supplementary material available at 10.1186/s11658-021-00284-7.

## Introduction

Ovarian cancer (OC) is the most fatal malignancy among women [1]. One in 70 will develop OC and its incidence rate based on standard age (ASR) is 66 per 1,000,000 (GLOBOCAN 2020) [2]. One in 100 women will die of the disease and the ASR of mortality is 39 per 1,000,000. OC accounts for 2.5% of all malignancies in women. Furthermore, 5% of deaths are due to the malignancy’s asymptomatic progression and low survival rates, which are mostly due to late stage-diagnosis [1, 3].

The incomplete understanding of the genetic alterations and molecular pathways of OC means treatments cannot be properly targeted. Thus, despite the relatively rapid development of diagnostic and therapeutic techniques, the prognosis for patients with OC is still unsatisfactory. Elucidating its pathogenesis could reveal novel and promising biomarkers and targeted therapy sites [4, 5].

Non-coding RNAs (ncRNAs) comprise more than 98% of the human genome. They are transcribed from DNA without being translated into a protein and play essential roles in gene translation and regulation [6]. Importantly, many studies have demonstrated the role of ncRNAs in the early stages of tumor development, with links to cell growth, metastasis, apoptosis, and invasion [7, 8].

Circular RNA (circRNA), a large class of non-coding regulatory RNA, was first discovered in 1976 [9, 10]. More than 30,000 circRNAs have already been discovered in human tissues [11]. They appear as circular genomes and exist in all kingdoms of life ranging from archaea to humans [12]. Most are expressed from protein-coding genes derived from single or multiple exons and are produced by back-splicing, a process that occurs in a reversed orientation. In other words, a downstream 5′-splice site is linked to an upstream 3′-splice site to produce a circRNA [13–15]. In recent years, circRNAs have been shown to be associated with various human disorders. Some have an important role in tumor onset and the progression of cancer. Genomic analysis shows a powerful presence of circRNA in many cancerous cell types [16–19]. Traits seen in these cancers are all present in OC. The potential roles of circRNAs in the pathogenesis of many human cancers have been confirmed through extensive studies conducted with novel extranuclear enrichment tools and next-generation sequencing (NGS), bioinformatics and microarray analyses, then validated for OC using quantitative real-time PCR with various primers [20]. Thus, it is known that many circRNAs are dysregulated in OC plasma, tissue and cell lines, and that they influence the development of OC by affecting cell proliferation, migration, invasion and apoptosis through absorption of miRNAs or impact on the cell cycle. Further research about circRNAs should focus on improving the potential of diagnosis and treatment of OC.

## Methods

This study focuses on the identification and expression of dysregulated circRNAs. It also assesses their importance in the diagnosis of OC and estimation of patient prognosis. The study was performed in accordance with the JBI methodology for systematic scoping reviews [21]. The proposed review title has been registered in JBI (https://joannabriggs.org/systematic-review-register) and its protocol has been registered on PRISMA flow diagram [22].

### Review question

Question: What role do circRNA expression levels play in patients with ovarian cancer (OC)?

### PCC

Participant type: Cisgender women with OC and related OC cell lines.

Concept: Circular RNA expression as biomarkers with prognostic and diagnostic functions.

Context: Not applicable and special.

### Search strategy

Based on the reporting objects used in systematic scoping reviews, our searches were performed using Web of Science, PubMed, Embase and Scopus. The keywords were "circular RNA", "CircRNA", "Ovarian Cancer" and "OC", as well as other synonyms for circRNAs. The latest date of publication was April 2021 and the publication language was English. The search results were combined and de-duplicated.

### Study screening and inclusion

EndNote X9 software was used to find and remove duplicates. References were arranged by number and exported to Microsoft Word for screening. The study process was summarized in a PRISMA flow diagram (Additional file 1).

### Study eligibility criteria

The eligible studies were included with the following criteria: studies evaluating the expression of circRNA profiling in patients with OC; the association between OC cell lines or human samples and circRNA; the potential of circRNAs as biomarkers for early diagnosis; and the connection between chemotherapy‐resistant metastatic OC and circRNAs. They had to be full-text articles in English. However, circRNA profiling research that uses cell lines or serum samples of patients with OC was also included. Those using other circRNA technologies, including NGS and PCR, those comparing OC tumor biopsies at various stages, and review articles were not encompassed.

### Data extraction

Reviewers extracted data from qualified studies. We specify information related to study identification (author, year of publication, number and location of centers, and journal), the list of circRNAs and the study design being expressed differentially. Related supplementary information, including samples or cell lines, the related mRNA, and their expressions, functions and potential targets were extracted from relevant online databases, including Circad, CircNet, CircBase, and PubMed (Table 1). The limitations of our study were that most of the included articles had incomplete demographic information, e.g., the stages and grades of OC were not given.

**Table 1:** CircRNA research databases

Name	URL
Circad	http://clingen.igib.res.in/circad/index.html
CircNet	http://circnet.mbc.nctu.edu.tw
CircBase	http://www.circbase.org
PubMed	https://pubmed.ncbi.nlm.nih.gov

### Data synthesis

**Fig. 1:**
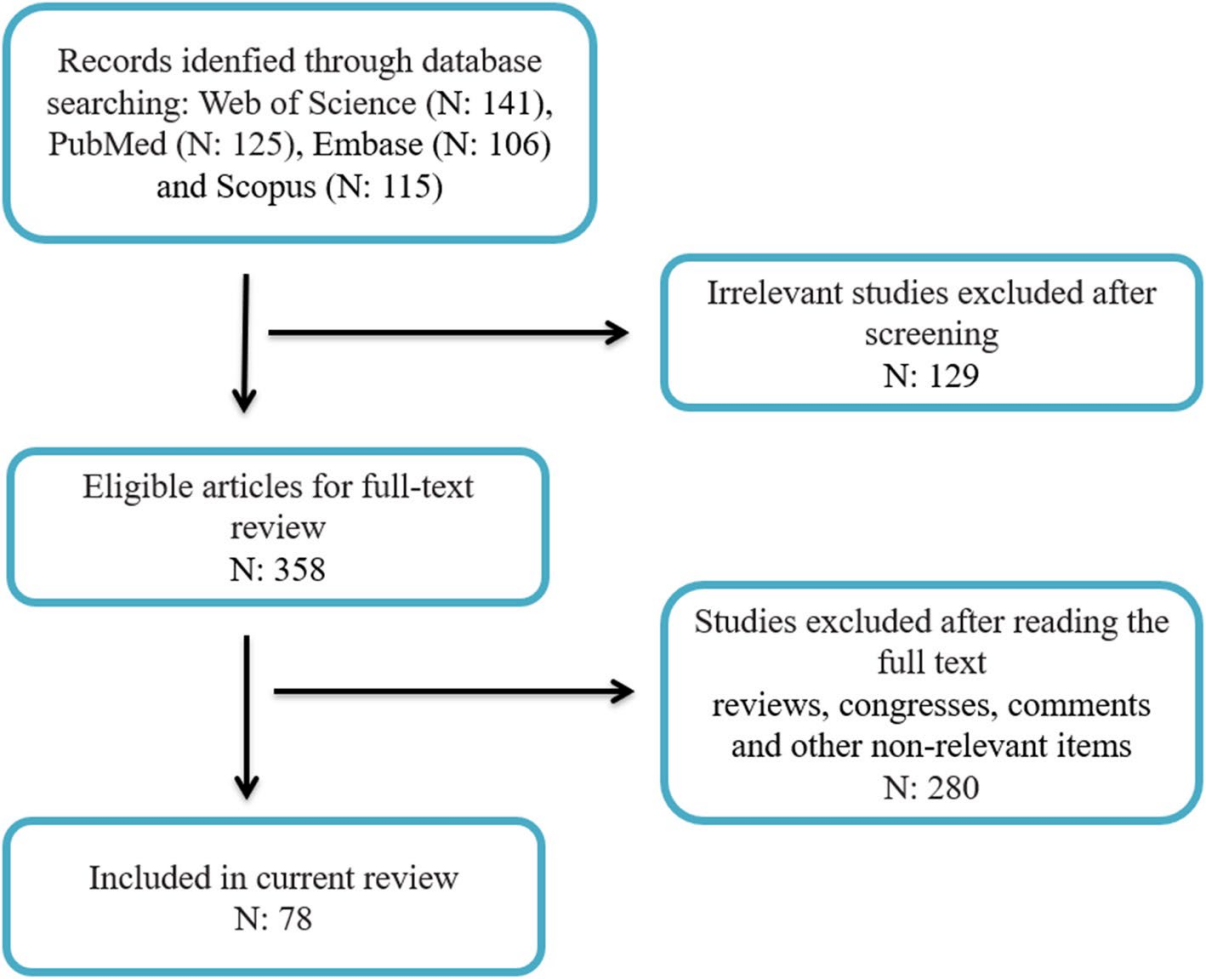
The flow diagram for the selection of studies

We extracted 487 articles from Web of Science, PubMed, Embase and Scopus. The results were merged and de-duplicated. After removing irrelevant articles, 78 abstracts were reviewed. The selection procedure is shown in the flowchart in Fig. 1. Data were organized based on circRNA name, targeted mRNA and its role in tumorigenesis (Table 2).

**Table 2:** Studies about dysregulated circRNAs in OC

Circular RNAs	Models or sample types	Regulation on expression	Targeted miRNA	Mechanism or effects on cancer progression	References
Circ-SETDB1	SOC patients and healthy volunteers	Upregulated	**–**	It may act as a novel biomarker for finding SOC improvement and predicting reaction to platinum-taxane-combined chemotherapy and recurrence in SOC	Wang et al. [73]
Circ-NFATC3	OC cell lines	Upregulated	–	It may play a promising role in increasing cell proliferation, migration and invasion in OC	Karedathet al. [121]
Circ-0015756	OC and adjacent normal tissues OC cell lines	Upregulated	miR‑942-5p	Suppressing Circ-0015756 inhibits OC promotion through the regulation of miR-942-5p/CUL4B pathway Circ-0015756 may serve as a novel therapeutic target for OC	Du et al. [88]
Circ-0002711	OC tissues and cells OC tissues and cell lines	Upregulated	miR-1244	Circ-0002711/miR-1244/ROCK1 pathway regulates tumor growth in vivo	Xie et al. [89]
Circ-0015326	Xenograft tumors in vivo	Upregulated	miR-127-3p	Circ-0015326 facilizes OC progression through absorbing and adjusting the miR-127-3p/MYB pathway Circ-0015326 may become a promising therapeutic target for OC	Zhang et al. [90]
Circ-0025033	OC tissues and cells	Upregulated	miR-330-5p	MiR-330-5p acts as a tumor suppressor in OC through targeting KLK4 Circ-0025033 may be a candidate in diagnosis and therapeutic target in OC	Cheng et al. [93]
Circ-0025033	OC tissues and cells	Upregulated	miR-184	Circ-0025033 accelerates the progression of OC through regulating LSM4 expression *by* absorbing miR-184 It provides a new therapeutic strategy for OC	Hou and Zhang [92]
Circ-MUC16	EOC tissues and healthy ovarian tissues Cell lines	Upregulated	miR-199a	Circ-MUC16 promotes autophagy of EOC through absorbing miR-199a and regulation of ATG13	Gan et al. [76]
Circ-KRT7	OC cell lines	Upregulated	miR-29a-3p	Circ-KRT7-miR-29a-3p-COL1A1 pathway increases tumorigenesis and promoting cell proliferation in OC	An et al. [139]
Circ-0072995	EOC tissues and cell lines	Upregulated	miR-147a	A novel diagnostic and therapeutic biomarker in OC It Enhances the progression of EOC via modulating miR-147a/CDK6 pathway	Ding et al. [31]
Circ-FGFR3	OC tissues and their adjacent normal tissues	Upregulated	miR-29a-3p	Regulating EMT of OC through miR-29a-3p/E2F1 pathway Its overexpression relates to poor prognosis in OC patients	Zhou et al. [83]
Circ-PGAM	EOC tissues and cell lines	Upregulated	miR-542-3p	Circ-PGAM1 accelerates malignant progression of EOC by modulation of the miR-542-3p/CDC5L/PEAK1 axis	Zhang et al. [90]
Circ-ABCB10	EOC and adjacent normal tissues EOC cell lines	Upregulated	miR-1271 miR-1252 miR-203	Circ-ABCB10 relates to advanced clinicopathological features and unfavorable survival It accelerates proliferation and reduce apoptosis through absorb of miR-1271, miR-1252 and miR-203 in EOC	Chen et al. [79]
Circ-ABCB10	EOC cells and normal epithelial ovarian cells	Upregulated	miR-1271	Circ-ABCB10 expression is associated with weak differentiation, progressive FIGO stage, and large tumor size by guiding the miR-1271-mediated Capn4/Wnt/β-catenin signaling pathway	Lin et al. [84]
Circ-HIPK3	EOC tumor tissues and adjacent noncancerous tissues	Upregulated	–	Promoting proliferation, migration and invasion and inhibit apoptosis	Teng et al. [32]
Circ-051239	EOC tissues and exosomes derived from plasma and cells Cell lines in vitro	Upregulated	miR-509-5p	Superior metastatic EOC cells can admit this potential to lower metastatic potential through exosomal Circ-051239, generating raised proliferative, migratory and invasive inclusions in receptor cells	Ma et al. [44]
Circ-EEF2	OC cell lines and In vivo tumor xenograft	Upregulated	miR-6881-3p	Circ-EEF2 increasing autophagy through absorbing miR-6881-3p and ANXA2 in EOC	Yong et al. [81]
Circ-RAB11FIP1	EOC and normal tissue	Upregulated	miR-129	Circ-RAB11FIP1 increasing autophagy of OC via DSC1 and miR-129 and may serve as a potential biomarker for EOC diagnosis and targeted therapy	Zhang et al. [82]
Circ-KIF4A	OC tissues	Upregulated	miR-127	Promoting OC progression via miR-127/JAM3 axis A promising novel biomarker and therapeutic target for OC	Sheng et al. [94]
Circ‑FAM53B	OC specimens and cell lines	Upregulated	miR‑646 miR‑647	Contributing to oncogenesis through sponging miR-646 and miR-647 leading proliferation, migration, and invasion in OC	Sun et al. [85]
Circ-Foxp1	Serum samples from EOC patients and healthy people	Upregulated	miR-22 miR-150-3p	Upregulated circulating exosomal Circ-Foxp1 is a self-determining prognosis of survival rate and drug resistance in EOC patients and can use as a novel biomarker and possible therapeutic target for EOC	Luo and Gui [107]
Circ-S-7	OC and normal adjacent tissues	Upregulated	miR-641	Circ-S-7 promotes OC EMT by mediating miR-641 to up-regulate ZEB1 and MDM2 pathways	Luo and Gui [107]
Circ-EPSTI1	OC tissues and cell lines	Upregulated	miR‐942	Circ-EPSTI1 adjusts OC promotion through decoying miR‐942 And can be serve as a biomarker and therapeutic target in OC	Xie et al. [89]
Circ-0061140	OC cell lines	Upregulated	miR-370	Regulating cell proliferation in OC via mediation of the miR-370/ FOXM1 pathway	Chen et al. [79]
VPS13C/Circ-001567	Tumor and adjacent tissues OC cell lines	Upregulated	–	Knocking down of VPS13C/Circ-001567, reduces cell invasion, cell cycle, cell proliferation and apoptosis ability	Bao et al. [66]
Circ-VPS13C	OC cells	Upregulated	miR-145	Its Upregulation in OC Accelerates the progression of OC Circ-VPS13C/miR-145/MEK/ERK axis suppressed with the treatment of Propofol	Lu et al. [67]
RAD50/ Circ-00718	Tumor and adjacent tissues OC cell lines	Upregulated	–	It takes part in OC development via adjusting the biological behaviors of OC	Bao et al. [66]
SPECC1/ Circ-000013	Tumor and adjacent tissues and OC cell lines	Upregulated	–	It takes part in OC development via adjusting the biological behaviors of OC	Bao et al. [66]
Circ-CSPP1	OC tissues and cell lines	Upregulated	miR-1236-3p	Increasing ZEB1 expression, EMT related markers, MMP-2 and VEGFA factors which are belong to tumorigenesis Promoting cell proliferation and invasion by sponging miR-1236-3p	Li et al. [61]
Circ-PIP5K1A	OC tissue and cell lines	Upregulated	miR-661	It contributes OC progression through targeting the miR-661/IGFBP5 pathway Associated with poor prognosis	Sun et al. [77]
Circ-CELSR1	OC tissue and cell lines	Upregulated	miR-1252	Increasing of Circ-CELSR1 is associated with enhanced drug resistance through Circ-CELSR1/miR-1252/FOXR2 axis	Zhang et al. [74]
Circ-CELSR1	OC tissue and cells	Upregulated	miR-149-5p	Reducing cell sensitivity in to drug resistance by regulating miR-149-5p/SIK2 pathway	Wei et al. [99]
Circ-GFRA1	OC tissues and adjacent normal tissues	Upregulated	miR-449a	It acts as a novel biomarker in prognosis and therapeutic targets	Liu et al. [55]
Circ-0013958	OC tissue and cell lines	Upregulated	–	Contributing OC promotion through affecting EMT and apoptosis Circ-0013958 may serve as a novel diagnostic and therapeutic target in OC	Pei et al. [71]
Circ‑0005585	Normal ovarian tissue Benign OC tissue Primary and metastatic EOC tissue	Upregulated	miR-23a miR-23b miR-15a miR-15b miR-16	ESRP1 overexpression was regulated by Circ-0005585 via sponging miR-15a, miR-15b, miR-16, and miR-23a, miR-23b, which caused inhibition of cancer cells migration and metastasis	Deng et al. [58]
Circ-102958	OC tissue and cell lines	Upregulated	miR-1205	Circ-102958 shows poor prognosis and promotes OC progression by miR-1205/SH2D3A pathway	Wang et al. [59]
Circ-MYLK	tumor tissue specimens and paracancerous normal ones	Upregulated	miR-652	Developing malignancy progression of OC through absorbing miR-652 associating with pathological staging and poor prognosis in OC patients	Zhao et al. [60]
Circ-PUM1	OC tissues Normal tissues Cell lines	Upregulated	miR-615-5p miR-6753-5p	It acts as a cancer derive exosome Promoting OC proliferation and migration	Guan et al. [72]
Circ-RhoC	OC tissues Normal tissues Cell lines	Upregulated	miR-302e	Accelerating tumorigenicity via positive regulation of miR-302e/VEGFA axis	Wang et al. [59]
Circ-0000714	OC cells Cell lines	Upregulated	miR-370-3p	Controlling and Regulating RAB17/CDK6/RB axis which progress in the malignancy of the paclitaxel-resistant OC cells	Guo et al. [100]
Circ-TNPO3	OC samples and correlated with PTX resistance	Upregulated	miR-1299	It plays a fundamental role in the chemoresistance and tumorigenesis of OC through upregulating NEK2 expression	Xia et al. [101]
Circ-NRIP1	Tumor xenograft models	Upregulated	miR-211-5p	It increasingly expresses in PTX-resistant OC tissues and cells Suppressing of Circ-NRIP, inhibited the PTX resistance in OC through modulating the miR-211-5p/HOXC8 pathway It may act as a prominent novel target in resistance therapy	Li et al. [102]
Circ-UBAP2	OC tissues and adjacent normal tissues	Upregulated	miR-144	Promoting OC progression via sponging miR-144	Sheng et al. [94]
Circ-UBAP2	OC tissues and cell lines	Upregulated	miR-382-5p	Promoting proliferation, migration and invasion and inhibit apoptosis	Xu et al. [95]
Circ-0001068	OC patients and healthy volunteers	Upregulated	miR-28-5p	Circ-0001068 was higher in OC patients than healthy ones Play as a prominent noninvasive biomarker and aim for the diagnosis and therapeutic targets in OC	Wang et al. [106]
Circ-NOLC1	OC tissues Normal tissues Cell lines	Upregulated	miR-326-5p miR-330 miR-370 miR-9-5p	Increasing cell growth, migration, invasion, and playing an oncogenic role by attaching ESPR1, RhoA, and modulating CDK1 levels	Chen et al. [108]
Circ-PVT1	EOC cell lines and Normal control cells	Upregulated	miR-149	Accelerating cell proliferation hindering apoptosis	Sun et al. [85]
Circ-PVT1	OC tissue and normal tissue	Upregulated	miR-149-5p	Increasing of PVT1 levels causes shorter survival rate in OC patients Increasing FOXM1 level through binding to miR-149-5p Causing OC cell viability and migration	Li et al. [86]
Circ-ASH2L	OC cells in vitro Tumor xenografts in vivo	Upregulated	miR-665	Acting as a serious role in adjusting OC cell tumorigenesis, lymphangiogenesis and angiogenesis by the miR-665/VEGFA pathway It is a thinkable candidate for therapeutic targets in OC	Chen et al. [79]
Circ-0051240	OC and corresponding adjacent non-cancerous tissues	Upregulated	miR-637	Boosting cell proliferation, migration and invasion in OC by miR-637/KLK4 pathway	Zhang et al. [82]
Circ-SMAD7	OC cells and tissue	Upregulated	miR-630	Circ-SMAD7/miR-630/KLF axis causing oncogenic behavior in OC	Zhao et al. [109]
Circ_0026123	OC cell lines and tissues	Upregulated	miR‑124‑3p	Increasing OC cells metastasis and proliferation via the miR‑124‑3p/EZH2 axis	Yang et al. [25]
Circ-WHSC1	normal ovaries and OC tissues	Upregulated	miR-145 miR-1182	Increasing expression of Circ-WHSC1 in OC promotes tumorigenesis via modulation of miR-145 and miR-1182	Zong et al. [64]
Circ-0009910	OC tissues Normal ovarian tissues OC cell lines	Upregulated	miR-145	Circ-0009910, representing poor prognosis in OC cells Inducing proliferation and motility through suppressing miR-145	Li et al. [65]
Circ-0002711 Circ-0001756	OC patient serum	Upregulated	–	These CircRNAs are presumably related to OC promotion, and may be promising novel biomarkers	Wang et al. [73]
Circ-0004390	OC tissue and normal tissue	Upregulated	miR-198	Circ-0004390 proliferation through miR-198/MET pathway, provides a therapeutic novel target for OC	Xu et al. [57]
Circ-1656	SOC tissues and ovarian cell lines	Downregulated	**–**	Acting as a promising biomarker and significantly associated with SOC FIGO stage	Gao et al. [112]
Circ-ITCH	Tumor tissues and paired adjacent tissues samples	Downregulated	–	Suppressing cells proliferation Increases cells apoptosis in EOC	Luo et al. [116]
Circ-ITCH	Human EOC cell lines human normal ovarian epithelial cell line	Downregulated	miR-10a-α	Suppressing cells proliferation Increases cells apoptosis in EOC	Luo et al. [116]
Circ-ITCH	OC tissues and cell lines	Downregulated	miR-145	Suppressing the malignancy of OC cells through the circ-ITCH-miR-145-RASA1 pathway	Hu et al. [113]
Circ-ITCH	OC tissues and cell lines	Downregulated	HULC-lncRNA	It’s downregulation of OC with the mediate of HULC lncRNA	Yan et al. [118]
Circ-9119	OC tissues and cell lines	Downregulated	miR-21	Regulating OC cells proliferation and apoptosis through absorbing miR-21 and targeting the PTEN–Akt axis	Gong et al. [120]
Circ-LARP4	OC tissue and adjacent normal tissue samples	Downregulated	–	It’s Low expression in OC patients, makes it a novel prognostic biomarker	Zou et al. [122]
CDR1as	OC and EOC tissues and cell lines	Downregulated	miR-135b-5p	By intermediation of miR-135-5p/ HIF1AN pathway, suppressing tumor formation	Chen et al. [131]
Cdr1as	OC tissues and cell lines	Downregulated	miR-1270	Reducing OC cells drug resistance by upregulation of SCAI and downregulation of miR-1270	Zhao et al. [109]
Circ-EXOC6B	EOC specimens and normal ovarian tissues	Downregulated	–	It can play a role as a potential prognostic biomarkers in patients with EOC	Ning et al. [110]
Circ-EXOC6B	OC cells in vitro OC cells in vivo	Downregulated	miR-376c-3p	Increasing sensitivity of drug resistance of OC cells Decreasing proliferation, migration and invasion of OC cells through miR-376c-3p/FOXO3 axis	Zheng et al. [114]
Circ-EXOC6B	Normal ovarian epithelial cells Human OC cell lines	Downregulated	miR-421	Promising target for the treatment of OC via sponging miR-421 and upregulating RSU1	Wang et al. [115]
Circ-BNC2	OC tissue and plasma samples	Downregulated	–	Potential diagnostic biomarkers in patients with EOC	Hu et al. [113]
Circ-RHOBTB3	OC cells in vitro OC cells in vivo	Downregulated	–	Suppressing cell proliferation and metastasis through PI3K/AKT signaling pathway	Hu et al. [113]
Circ-PLEKHM3	Primary OC/metastatic OC/normal ovarian tissues/cell lines	Downregulated	miR-9	Targeting miR-9/BRCA1/DNAJB6/KLF4/AKT1 axis Inactivates the PI3K/AKT and Wnt/catenin pathways via promoting BRCA1, DNAJB6a, and KLF4 expression by sponging miR-9	Zhang et al. [123]
Circ-0007444	OC patients and OC cells	Downregulated	miR-570-3p	Circ-0007444 suppressing OC promotion through the miR-570-3p/PTEN pathway It may serve as a candidacy for targeted therapy	Wu et al. [140]
Circ-ANKRD12	OC cell lines	Downregulated	miR-4768-5p	Expression of Circ-ANKRD12, suppresses invasive molecular behaviors and phenotypes Circ-ANKRD12 can act in a various set of tasks ranging from cell cycle arrest, tumor invasion to immune incorporation	Karedath et al. [121]
Circ-0078607	OC tissues and adjacent tissues OC cell lines	Downregulated	miR-518a-5p	Circ-0078607 downregulation, inhibits OC progression through miR-518a-5p/Fas axis	Zhang et al. [123]
Circ-0007874	OC cell lines	Downregulated	miR-760	Circ-0007874 downregulation, inhibits OC progression through miR-760/SOCS3 pathway	Li et al. [125]
Circ-MTO1	OC tissues and adjacent tissues OC cell lines	Downregulated	miR-182-5p	Circ-MTO1 suppresses the proliferation and invasion of OC cells via the miR-182-5p/KLF15 pathway	Wang et al. [115]
Circ-100395	OC tissues	Downregulated	miR-1228	Circ-100395 suppresses cell proliferation and metastasis of OC cells through regulating the miR-1228/p53/EMT pathway	Li et al. [125]

## Discussion

As with other non-coding RNAs (ncRNAs), circRNAs can adjust gene expression by sponging protein or RNA. The most-reported way involves regulating miRNA activity by acting as competition for endogenous miRNA sponge elements. This allows mRNA to escape from miRNA suppression post-transcriptionally [23]. The size of circRNAs ranges from several hundred to thousands of nucleotides that are produced from one to five exons. Since they do not have a free end, they are not prone to exonuclease degradation, making them very stable. They primarily reside in the cytoplasm or nucleus, originating from the precursor mRNA through back splicing of exons or introns (cytoplasmic) or simple splicing (nucleic) [24]. They can be categorized into three types based on differences in their genomes and main sequences: circular intronic RNAs (ciRNAs), exonic circRNAs (ecircRNAs), and exon–intron circRNAs (EIciRNAs) [25].

**Fig. 2:**
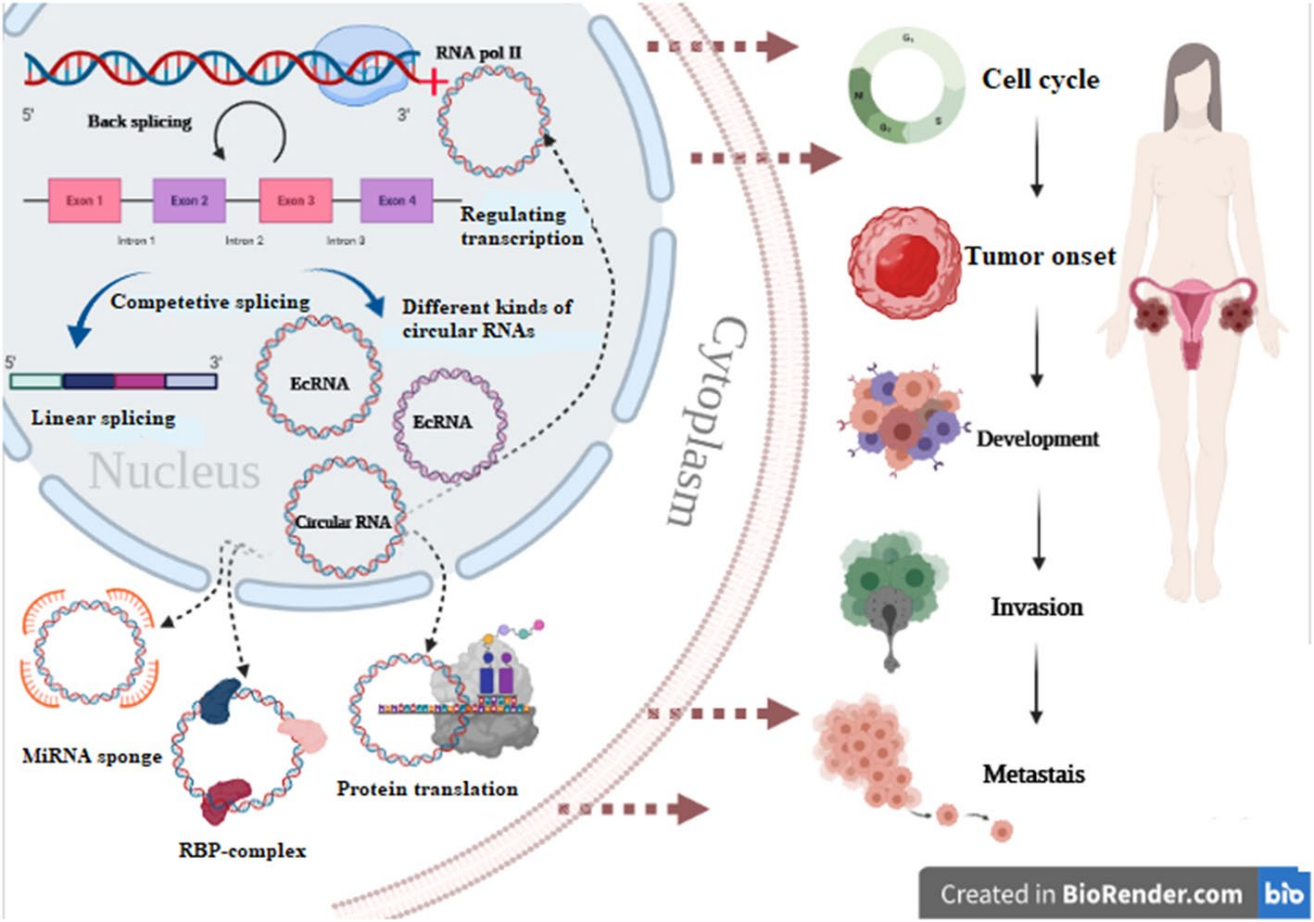
The various functions of circRNA

Notable characteristics of circRNAs include their prevalence and specificity [23, 24], high stability [26] and conservation [27]. They qualify as valuable clinical biomarkers or targets. Multiple biological roles for circRNAs have been explored, including:

1. CircRNAs are specific miRNA reservoirs or sponges.
2. CircRNAs interact with RNA-binding proteins (RBPs), which play a central function in transcription and translation of genes.
3. CircRNAs act as protein or peptide translators.
4. CircRNAs act as regulators of gene transcription and expression (Fig. 2).

Considerable evidence has shown that circRNAs can affect the aggressive properties of tumors. For example, they compete with miRNAs, translate into proteins, act as miRNA reservoirs, and form fusion circRNAs (f-circRNAs) [28, 29]. Their unique characteristics, such as tissue-specificity, diversity and high stability could make them useful diagnostic markers, provided we develop a better understanding of their expression in cancer tissues [30].

According to the information we have about the importance of these circRNAs, their effect on various organs is not negligible. One of the organs that can be affected by these circRNAs is the ovaries. Ovaries play a fundamental role in the female reproductive system with the basic functions of producing oocytes under the stimulation of gonadotropic hormones secreted from the pituitary gland to guarantee the evolution of follicles, estrous cycles, and post-pregnancy hormonal levels. The risk factors for OC include age, a family history of the disease, and unremitting ovulation in carriers of the heredity mutation of BRCA1 [31]. The occurrence and progression of OC is a complicated process, encompassing multi-factor, multi-step and multi-gene regulation [32]. OC comprises a group of heterogeneous malignancies recognized via cell or site of origin, risk factors, pathological degree, prognosis, and treatment. It has three principal types: epithelial, germ cell, and sex-cord-stromal, with the latter two containing just about 5% of all OCs [33]. The most common malignancies amongst women of all ethnicities and groups are epithelial cancers, counting for 90% of all cases [34]. There are four primitive histological subgroups of epithelial ovarian cancer (EOC): serous, endometrioid, mucinous, and clear cell [35]. Serous tumors are classified into two categories: high-grade serous carcinomas (HGSC) or low-grade serous carcinomas (LGSC) [36].

Invasive therapies, such as surgery, and drug therapies have not sufficiently reduced OC mortality. Finding new biomarkers and targets may help with earlier diagnosis and more effective treatment. The relationships between circRNAs and OC have considerable potential because of their regulatory roles. It is known that abnormalities in the regulation of signaling pathways and cellular mechanisms cause cancer formation, progression, and metastasis. In the past few years, multiple studies have focused on differentially expressed circRNAs in OC, indicating that circRNAs could be therapeutic targets and novel biomarkers for this disease [37].

### CircRNAs in cancer

Various internal and external factors are involved in cancer pathogenesis. Genetic and epigenetic factors are the main ones [38, 39]. Multiple recent studies have confirmed that circRNAs play a substantial role in the initiation and progression of various cancers, such as hepatic and renal carcinoma, and bladder, prostate, esophageal, breast, gastric and lung cancer [40–45]. Considerable evidence shows that these circRNAs function in a great number of cancers and play essential roles in their occurrence and development [46]. However, the results of studies on cancer cell lines and tissues indicate that circRNAs can be involved as oncogenes or tumor-suppressor genes [47, 48].

### CircRNAs and their roles in OC pathogenesis

Ahmed et al. were the first to conduct an expression analysis on circRNAs in ovarian tumors. They found that a great number of circRNAs are differentially expressed in tumors [49]. They also examined the differential expression of circRNA and mRNA between primary and metastatic ovarian tumors. Because of the stability of circRNAs against exonuclease resistance [50], they could recognize the primary tumor from metastatic lesions. Many circRNAs have been identified as oncogenes by focusing on circRNA–miRNA sponging and recognizing the biological effects and regulatory networks involving the miRNAs and the subsequently targeted genes. Multiple circRNAs have an oncogenic pattern in OC since their upregulation boosts cell progression and tumor growth. Based on the most recent evidence from CircBase and Interactome analysis, circ-NFATC3, which is involved in ovarian cancer, can potentially adjust multiple biological operations: protein decoy, cell proliferation and motility, apoptosis, senescence, and cell responses to oxidative stress. It achieves its effects through post-transcriptional alignments, like cell translation, conservation and RNA alternative splicing, via sponging RBPs and microRNAs [51].

Multiple circRNAs, such as circ-0051240, circ-EPSTI1, circ-PIP5K1A and circ-GFRA1, cause malignant behavior of OC cells by acting as miRNA sponges [52–55]. circ-PIP5K1A becomes overexpressed when miR-661 is targeted. Circ-PIP5K1A is located on chromosome 1. Silencing it markedly suppressed the ability of OC cells to proliferate and invade other tissues [54]. Xie et al. showed the remarkable upregulation of circ-EPSTI1 in OC cells and specimens in comparison with their adjacent non-tumor counterparts. Based on their observation, the inhibition of circ-EPSTI1 can suppress cancer cell proliferation and migration, which causes apoptosis in OC. This emphasizes the carcinogenic role of this circRNA [53].

Circ-UBAP2 promotes the expression of N-cadherin in OC cells by sponging miR-144 as well as exerting conservative effects on OC cell proliferation and invasion. Overexpression of miR-144 has a reverse effect on Circ-UBAP2 tumorigenesis [56]. Another study conducted on circ-UBAP2 showed that cell proliferation increases upon overexpression of circ-UBAP2 and cell apoptosis decreases via the miR-382-5p/PRPF8 pathway [57]. Deng et al. found that ESRP1 overexpression is regulated by circ-0005585 via sponging of miR-15a, miR-15b, miR-16, miR-23a and miR-23b, which inhibited cancer cell migration and metastasis through alternative splicing of two cytoskeleton-associated proteins: EPB41L5 and Rac1 [58].

Silencing circ-102958 significantly inhibits OC cell proliferation and invasion and its upregulation is associated with a poor prognosis in OC patients. It promotes OC cell proliferation and migration by increasing the expression of SH2D3A through influence on the activity of miR-1205 as a competing endogenous RNA (ceRNA) [59].

The circ-MYLK level in patients with OC has been found to be significantly higher in cancer tissues than that in adjacent ones. Circ-MYLK can be targeted by microRNA-652 [60]. Li et al. assessed the mechanism of another oncogenic circRNA, circ-0001806 (also named circ-CSPP1), which is located at position 13.2 on the long (q) arm of chromosome 8 and derived from centrosome/spindle pole-associated protein 1 gene. This circRNA was found to be upregulated in OC tumors and tumor margins. However, an increase in miR-1236-3p due to silencing circ-CSPP1 in OC cell lines, decreases ZEB1 expression at the RNA and protein levels [61].

Hsa-Circ-0026123 inhibits OC progression by mediating the miR‑124‑3p/EZH2 candidate biomarker for the diagnosis and therapeutic target of OC [62]. In 2019, researchers found circ-SMAD7 on chromosome 18 was upregulated and promoted tumorigenesis in OC cells. Using bioinformatics procedures, a tumor suppressor protein, Krüppel-like factor 6 (KLF6), was proven to be highly downregulated in OC tissues. Increased expression of circ-SMAD7 might result suppress KLF6, leading to tumor formation [63]. Microarray screening of circ-WHSC1, which is more highly expressed in OC cells than normal ones, revealed that the linkage of miR-1182 and miR0145 promotes tumor formation by increasing cell proliferation and metastasis in OC [64]. Circ-0009910 is an oncogenic circRNA indicative of poor prognosis in patients with OC. It induces tumor growth via interaction with miR-145: together, they influence the JAK1/STAT and NOTCH signaling pathway to accelerate proliferation and metastasis [65]. Bao et al. investigated VPS13C/hsa-circ-001567, RAD50/hsa-circ-00718 and SPECC1/hsa-circ-000013 to establish their vital role in OC [66]. They found that the expression of these three circRNAs was markedly increased in tumor tissues in comparison with non-cancerous samples. VPS13C/hsa-circ-001567 had the highest expression in the tumor tissues. Propofol has been used to control and tune tumor cell growth with the dual-luciferase assay used to monitor the outcomes. Propofol treatment downregulated circ-VPS13C, suppressing the activation of the MEK/ERK signaling pathway via upregulation of miR-145 [67].

Multiple studies are showing that circRNAs are significant in EOC. Circ-PGAM1 was found to be an oncogenic circRNA in EOC tissues and cells by Zhang et al. They silenced it and showed inhibition of progression in EOC tissues and cells. A related miRNA, miR-542-3p, was found to be downregulated in EOC tissues and cells. Its overexpression suppressed the formation of tumors in related tissues and cells [68].

As mentioned before, circRNAs have other functions and can play a crucial role in tumor cell proliferation, apoptosis, metastasis and invasion. Here are some of the studies that investigate possible mechanisms by which circRNAs take part in OC development. Chen et al. found high distribution of circ-ASH2L in the cytoplasm, where it competes with vascular endothelial growth factor A (VEGFA) to absorb miR-665. This evidence justifies the role of circ-ASH2L in tumorigenesis and angiogenesis via the miR-665/VEGFA pathway [69]. The incidence of epithelial–mesenchymal transition (EMT) was noted as one of the factors of tumor metastasis. Circ-KRT7 increases EMT-related cell progression through sponging miR- 29a-3p in OC. This study suggests this biological role could be a factor for the diagnosis and treatment of OC [70]. Circ-0013958 also contributes to the development of OC: it shows high expression in OC cells and tissues, and it affects EMT and signaling pathways related to apoptosis. Although circ-0013958 inhibition can increase the amount of cell apoptosis, it can suppress OC cell growth, migration and metastasis in vitro [71].

There is a high expression of circ-PUM1 in normal ovarian tissues. There is also a direct relationship between its high expression and advanced stages of OC as defined using the FIGO scale from the International Federation of Gynecology and Obstetrics. Cell line-based studies revealed that circ-PUM1 increases cell invasiveness and proliferation by sponging miR-6753-5p and miR-615-5p to upregulate MMP2 and NF-κB expression. Guan et al. found that Circ-PUM1, on the one hand, causes rapid OC cell growth, invasion and metastasis, and on the other hand, by acting on the peritoneum, contributes to cancer metastasis via the formation of cancerous exosomes [72].

Many circRNAs, such as circ-RhoC, promote angiogenesis in OC. High expression of circ-RhoC promotes tumor formation and metastasis. However, this circRNAs sponging of miR-302 upregulates VEGFA expression, which is involved in metastasis and angiogenesis [73].

Zhang et al. discovered that one of the functions of circRNAs is to attenuate the inhibitory effects of miRNAs on protein translation by competing with them and interfering with target mRNAs. As an example, circ-S-7 has 74 conserved binding sites that can attach to the targeted miRNA. Meanwhile, it can also interact with miRNAs by attaching to argonaute proteins. Circ-S-7 expression is significantly higher in OC tissues and cells, indicating an association with the lymph node metastasis status, tumor node metastasis (TNM) classification of malignant tumor stages, and overall survivorship rates in patients with OC. Its silencing inhibited the growth and metastasis of OC cells [74].

Circ-NOLC1 is another circRNA that is more highly expressed in EOC tissues than in normal tissues and is positively related to the FIGO-defined stage. Its overexpression in cell lines is proven to increase cell growth, migration and invasion. It plays an oncogenic role by attacking to ESPR1 and RhoA, and by modulating CDK1 levels [75].

The next circRNA with diagnostic potential in EOC patient serum and tissues is circ-0049116, which is generated from a cell surface-associated protein named mucin 16. Increased expression of the circ-MUC16/miR-199a-5p axis positively correlates with the degree of tumorigenesis in EOC patients [76]. The expression levels of circ-FAM35b, circ-051239, circ-ABCB10, circ-0072995, circ-EEF2, circ-RAB11FIP1, circ-FGFR3, circ-NOLC1 and circ-PGAM1 correlate with diverse clinical and pathological traits of EOC [68, 75, 77–83]. To determine the potential role of the molecular mechanism of circ-FAM35B besides its prognostic ability in OC, a luciferase reporter gene assay and bioinformatics analysis were done. The results showed its overexpression correlated with proliferation, metastasis and prohibition of apoptosis in OC samples [77]. Superior metastatic EOC cells possess the capability to lower metastatic potential through exosomal circ-051239, leading to more proliferative, migratory and invasive inclusions in receptor cells [78].

Of all the circRNAs known to be involved in EOC, circ-ABCB10 is associated with the worst overall survival rate (OS). According to the in vitro experiments done by Chen et al., circ-ABCB10 showed negative regulation of miR-203, miR-1271 and miR-1252 in EOC cells as well as an association with increased cell proliferation and decreased apoptosis rate. Thus, circ-ABCB10 expression is associated with weak differentiation, progressive FIGO stages, and large tumor size. This is known to be due to its guiding of the mir1271-mediated Capn4/Wnt/β-catenin signaling pathway [79, 84].

Circ-0072995 increases malignancy through its influence on the miR-147a/CDK6 axis. Circ-EEF2 and circ-RAB11FIP1 facilitate autophagy by sponging mir-6881-3p and miR-129 in EOC [80–82]. Another oncogenic circRNA affecting EOC is plasmacytoma variant translocation 1 (PVT1), which is an extremely dysregulated gene in malignancy and is reported to have an association with EOC oncogenesis [85]. Although circ-PVT1 increases cell proliferation, it decreases apoptosis by sponging miR-149. It is therefore a potential a treatment target in EOC [86]. The alteration of cell viability and drug resistance was found with the intervention of FOX transcription factors (FOXM1) with the involvement of the PTV1/miR-149-5p axis.

### Roles in clinical implications of OC

Some CircRNAs have potential as biomarkers because their abnormal regulation is associated with pathological and clinical outcomes in OC patients. In 2020, Wang et al. conducted a comprehensive study using the serum of patients with OC instead of tissue samples. Using bioinformatics analyses of data and studying miRNA-binding sites, they identified 5 important upregulated circRNAs: circ-0002711; Chr5:170610175-170632616+; circ-0001756; Chr4:147227078-147230127-; and Chr16:53,175091-53191453+. They all showed an association with dysregulation in OC and were considered potential diagnostic biomarkers [87].

Numerous studies have shown that circ-0015756, circ-0002711, hsa-circ-0015326, circ-0001068, circ-0025033 and circ-KIF4A are oncogenic circRNAs with diagnostic and prognostic value in OC [88–94]. Their function occurs through sponge of corresponding miRNAs. Circ-0015756 increases the progress of OC by sponging miR‑942‑5p and influencing the miR‑942‑5p/CUL4B axis [88]. Similarly, the circ-0002711/miR-1244/ROCK1 and has-circ-0015326/miR-127-3p/MYB pathways are key players in OC with potential as therapeutic targets [89, 90]. Outcomes from a larger cohort showed that circ-0001068/miR-28-5p in T cells induced PD1 expression, and this caused in T-cell exhaustion [91]. Circ-KIF4A and circ-0025033 promote OC progression by absorbing miR-127 and miR-184, respectively [92, 94]. In 2020, another study was conducted on circ-0025033 that confirmed its role in cell viability, invasion [93]. These alterations occur through the miR-330-5p/KLK4 axis. This regulatory mechanism confirms circ-0025033 may be a prominent therapeutic target for OC.

Circ-0004390, which is derived from LPAR3 gene, is significantly upregulated in OC cells and tissues. Its knockdown can markedly reduce OC cell proliferation. By sponging miR-198, circ-0004390 increases cell proliferation to regulate the expression of hepatocyte growth factor receptor in OC cells. Xu et al. expressed that the level of circ-0004390 was closely related to the overall survivor level in patients with OC and found that it regulates OC proliferation via miR-198. The axis provides an important target in therapy [95].

One of the most valid and effective treatments for patients with cancer is chemotherapy. However, the chemical resistance resulting from the constant use of this method has gradually become a concern. In recent years, circRNAs have been considered significant targets and markers for the prognosis, diagnosis and treatment of many diseases, especially cancer [96]. In 2012, circ-0061140 was detected using luciferase reporter assays and RNA fluorescence in situ hybridization (FISH) technique and was analyzed using bioinformatic techniques. The results show the promotion of cell proliferation and metastasis in cell lines because of circ-0061140 sponging miR-370, which was followed by a decrease in FOXM expression [97]. In addition to circ-0061140, FOX transcription factors also cooperate with circ-CELSR1. Two separate molecular assay-based studies were conducted on circ-CELSR1 and its potential role in drug resistance (PTX-resistance) [98, 99]. Circ-CELSR1 overexpression was determined in PTX-resistant cells and tissues. Using quantitative real-time PCR and microarray analyses, Zhang et al. found that circ-CELSR1 was associated with an aggressive OC phenotype and acted through sponging miR1252. Indeed, the circ-CELSR1/miR-1252/FOXR2 axis directly effects on paclitaxel-resistance (PTX) [98]. The next study on circ-CELSR1 was done on murine xenografts. The role of the circ-CELSR1/miR149-5p/salt inducible kinase 2 (SIK2) axis as an essential regulator in drug resistance was proven [99].

Three other studies affirmed a high expression of circRNAs in PTX-resistant cells and tissues in OC patients [100–102]. These circRNAs, has-circ-0000714, circ-TNPO3 and circ-NRIP1, perform their functions as oncogenes in cells or tissues by sponging miRNAs and affecting molecules or signaling pathways. Hsa-circ-0000714 targets the CDK6/RB signaling pathway through regulation of RAB17. It acts as a miR-370-3p sponge, authorizing its regulation of RAB17 expression. Finally, these functions cause an increase in malignancy due to PTX-resistance in OC cells [100]. Similarly, circ-TNPO3 (has-circ-0001741) sponges miR-1299 and plays fundamental roles in the chemoresistance and tumorigenesis of OC through upregulation of NEK2 expression [101].

The findings of Li et al. provide novel information for overcoming chemotherapy resistance in OC. They confirmed the inhibitory mechanism of miR-211-5p against drug resistance in PTX-resistant OC cells. However, circ-NRIP1 hinders this action by sponging miR-211-5p and decreasing its expression. Simultaneously, HOXC8 expression increases and leas tumorigenesis [102].

As mentioned above, EOC is the most significant pathological subtype of OC, accounting for more than 90% of cases. Amongst the subtypes of EOC, SOC (high-grade serous ovarian cancer) accounts for 60–70% [103]. Some evidence indicates the potential role of circRNAs as serum biomarkers in several cancers, including these [104, 105]. For instance, circSETDB1 is upregulated in the serum and positively correlated with lymph node metastasis and developed clinical-stage in SOC [106]. Many studies have stated that circRNA expression is higher in EOC tissues than in adjacent normal tissues. Circ-HIPK3 is one of the best examples of this. In 2018, Ning Liu et al. showed that higher circ-HIPK3 expression is associated with poor prognosis of EOC patients, indicating that circ-HIPK3 can act as an important biomarker for prognosis of EOC [32]. Further studies done using RT-qPCR demonstrate the role of circ-FGFR3 as a suitable prognostic biomarker in EOC. Overexpression of circ-FGFR3 through sponging miR-29a-3p and consequently upregulation of E2F1 induced EOC cell EMT in vitro [83]. Circ-Foxp1 is a circulating exosomal RNA that could be a biomarker and potential remedial target for EOC. Its knockdown can decrease cell proliferation and increase drug resistance sensitivity [107].

### CircRNAs as a tumor suppressor

There is a group of circRNAs that play inhibitory roles in OC tumorigenesis and thus have tumor-suppressive effects. Cancer cells have the ability to avoid anti-growth signals via suppression of the expression of tumor suppressor genes. The anti-growth signals are key through arresting of the cell cycle. These circRNAs, like oncogenic ones, sponge miRNA to manage the malignancy of OC cells. CDR1as may be the best example of a tumor-suppressive circRNA [108, 109]. Its overexpression inhibits the proliferation, migration and invasion of OC cells. The mechanism involves miR-135B-5P. Silencing CDR1as increases the expression of miR-135B-5P, which in turn reduces the expression of hypoxia-inducible factor 1-alpha inhibitor (HIF1AN), thus increasing proliferation capacity in OC cells [108]. A separate study conducted on the suppressor effect of CDR1as demonstrated that its expression in OC cisplatin-resistant cells was severely downregulated [109]. The authors found that increasing Cdr1as could promote the sensitivity of OC cells to cisplatin, with the effect occurring through regulation of the miR-1270/SCAI axis.

Circ-BNC2, circ-EXOC6B, circ-FAM13B, circ-N4BP2L2, circ-RHOBTB3, circ-CELSR1, circ-ITCH and circ1656 are involved in EOC in a tumor-suppressive capacity. They could also potentially act as novel diagnostic biomarkers [110–112]. Circ-BNC2 is already used as a diagnostic marker in patients with OC. It is downregulated in both serum and tissues [113]. Circ-EXOC6B and circ-N4BP2L2 are known as potential prognostic biomarkers in EOC. CircEXOC6B affects drug resistance, inhibiting the PTX-resistance and progression of OC cells by detaching miR-376c-3p, which increases the FOXO3 level [114]. Wang et al. also confirmed circ-EXOC6B’s inhibitory role in the progression of OC, establishing the promotion of proliferation and invasion of OC cells through circEXOC6B downregulation [115].

It has been shown that the expression of Circ-ITCH decreases in EOC cell lines in comparison to normal ones and is associated with tumor size, decreasing FIGO stage, and inhibition of cell proliferation [111]. However, it was also found that overexpression of this circRNA extensively suppresses cell proliferation and apoptosis through an interaction with miR-10a [116]. Another study performed on circ-ITCH found an association with poor prognosis, but the inhibition of tumor progression could be accomplished via the circ-ITCH/miR-145/RASA1 signaling pathway with an increase in the expression of RASA1 in OC cells and tissues in both in vivo and in vitro. Downregulation of circ-ITCH inhibits OC cell growth, migration and invasion [117]. Yan et al. also confirmed circ-ITCH downregulation in OC [118].

Using RT-qPCR, Yalan et al. established that circ-RHOBTB3 downregulation induces OC formation with the inactivation of the PI3K/AKT signaling pathway [119]. Another tumor suppressor circRNA, Circ-9119, was found to inhibit cell viability by influencing the PTEN/AKT signaling pathway through a competitive reaction with miR-21 as a prognostic and therapeutic factor in OC [120].

Circ-ANKRD12 locates in the cytoplasm and is a potential clinical biomarker. It is involved in various functions, including invasion, cell cycle arrest, cancer metabolism alteration, and immune system modulation [121]. The expression of circ-LARP4 significantly decreases in OC tissues and it can act as a potential biomarker for prognosis [122]. Circ-PLEKHM3 is another downregulated circRNA found in OC cells. It sponges miR-9 and inactivates the Wnt/β-catenin and AKT signaling pathways, suppressing OC cell growth and migration [123].

Like other tumor suppressor circRNAs, circ-0078607 downregulation leads to OC formation. Zhang et al. indicated that inducing Fas expression via oncogenic miR-518a-5p suppresses OC. This circRNA is suggested as a new effective therapeutic target in OC treatment [124]. Circ-0007874 also provides a new therapeutic approach for OC with its tumor-suppressive potential through regulation of the miR-760/SOCS3 axis [125].

Circ-MTO1/miR-182-5p/KLF15 also inhibits OC progression [126]. Circ-100395 showed downregulation in OC and poor prognosis value. It inhibits cell proliferation and metastasis of OC cells by regulating the miR-1228/EMT/P53 pathway [127]. Two recent studies on Circ-0007444 and has-Circ-0026123 highlighted their suppressive roles. The circ-0007444/miR-570-3p/PTEN axis can be a candidate for target therapy [120].

### CircRNA biogenesis

In recent years, broad research on circRNA biogenesis has been conducted. At first, it was believed that circRNAs were just a transcriptional derangement from the RNA splicing process, but further study has fully authenticated their strictly regulated biosynthesis. However, that biogenesis is not yet fully elucidated, leaving more space for research. It is currently known that circRNAs are single-stranded, looped RNA molecules that are generated from pre-mRNAs via a back-splicing process expressed under particular situations. They principally reside in the cytoplasm where they have various functions associated with binding to other molecules such as proteins and miRNAs [128].

## Conclusion

Mounting evidence shows that circRNAs are dysregulated in cancerous tissues and can intercede in diverse signaling pathways, resulting in tumorigenesis, invasion and metastasis [129]. Impairment of some circRNAs in OC cell lines and tissues increases cancer progression by inducing cell division, migration and invasion [130]. CircRNAs can participate in the regulation of tumorigenesis in different types of malignancies using their regulatory structures. The improved understanding of circRNAs helps elucidate the molecular mechanisms involved in OC. Furthermore, because of their high stability and ubiquitously presence in body fluids, including breast milk, saliva, urine and blood, and in membrane vesicles, such as exosomes, circRNAs may serve as promising biomarkers for the early diagnosis and therapeutic achievements in cancer [131].

## Outlook

One of the most important needs in medical science is to have methods for the early detection and non-invasive treatment of cancer. CircRNAs have attracted considerable attention. Thanks to the rapid development in the fields of biotechnology and bioinformatics data analysis, a large number of these RNAs have been identified in different organisms. Due to their unusual expression and their effect on cancer cell growth, proliferation, apoptosis and metastasis, they can be an appropriate option for use as diagnostic biomarkers and therapeutic targets [132, 133]. To the best of our knowledge, the presence of circRNAs in exosomes in cellular communications [134] can be significant for early diagnosis and prognostic determinations [135].

Although sponging miRNAs is a substantial function of circRNAs, many do not have binding sites for miRNAs. Therefore, there may be other means of action besides sponging miRNAs. More studies are needed to elucidate the relationships and pathways relevant for miRNAs, circRNAs and proteins.

Many recent studies into circRNAs and their role in tumor resistance to chemotherapy indicate the involvement of regulatory pathway mechanisms, such as EMT processing, apoptosis, ceRNA regulation and autophagy [136]. Although many advances have been made in the field of circRNAs in OC, especially in the field of drug resistance and chemotherapy resistance, it is currently not possible to apply this knowledge fully in clinical procedures [137]. It is vital to recognize an effective therapeutic target that can tenderize OC to drugs and clarify the molecular pathway of drug resistance in this malignancy. Subsequent findings about circRNAs should also be explored. Some novel technologies, like nanopore sequencing, can potentially gather data on the whole circRNA and be a significant addition to the transcriptome toolbox for mammalian studies [138].

All the descriptions in this paper form just a small part of the capabilities and potential of these unique molecules in the prognosis and diagnosis of OC. Further comprehensive studies are needed to decipher the numerous aspects of circRNA behavior and use them in future clinical procedures.

## Supplementary Information

**Additional file 1.** PRISMA Checklist

## Data Availability

The data that support the findings of this study are available on request from the corresponding author. The data are not publicly available due to privacy or ethical restrictions.

## Abbreviations

OC: Ovarian cancer
EOC: Epithelial ovarian cancer
HGSC: High-grade serous carcinomas
LGSC: Low-grade serious carcinomas
CircRNAs: Circular RNAs
MiRNA: MicroRNA
NcRNAs: Non-coding RNAs
CeRNA: Competing ENDOGENOUS RNA
Pre-mRNA: Precursor mRNA
AUC: Area under the receiver operating characteristic curve
RT-qPCR: Reverse transcriptase-quantitative real-time PCR
FIGO: International Federation of Gynecology and Obstetrics
EMT: Epithelial–mesenchymal transition
CDR1as: Cerebellar degenerative-relate protein-1
FOXM1: Forkhead box M1
CSPP1: Centrosome/spindle pole-associated protein 1
GFRA1: GDNF family receptor alpha 1
EPSTI1: Epithelial stromal interaction 1
IGFBP5: Insulin-like growth factor-binding protein-5
ZEB1: Zinc finger E-box binding homeobox1
HIF1AN: Hypoxia-inducible factor-1 alpha inhibitor
ITCH: Itchy E3 ubiquitin protein ligase
RASA1: RAS P21 protein activator 1
HIPK3: Homeodomain-interacting protein kinase 3
ABC Transporters: The ATP-binding cassette transporters
NOLC1: Nucleolar and coiled-body phosphoprotein 1
CDK1: Cyclin dependent kinase 1
ESRP1: Epithelial splicing regulatory protein 1
RhoA: Ras homolog family member A
ABCB10: ATP binding cassette subfamily B member 10
FAM53B: Family with sequence similarity 53 member B
CELSR1: Cadherin EGF LAG seven-pass G-type receptor 1
PTX- Resistance: Paclitaxel resistance
VPS13C: Vacuolar protein sorting 13 homolog C
PVT1: Plasmacytoma variant translocation 1
